# Investigation of Self-Assembly Processes for Chitosan-Based Coagulant-Flocculant Systems: A Mini-Review

**DOI:** 10.3390/ijms17101662

**Published:** 2016-09-30

**Authors:** Savi Bhalkaran, Lee D. Wilson

**Affiliations:** Department of Chemistry, University of Saskatchewan, 110 Science Place, Saskatoon, SK S7N 5C9, Canada; sab589@mail.usask.ca

**Keywords:** arsenic, coagulant, flocculant, chitosan, metal salts

## Abstract

The presence of contaminants in wastewater poses significant challenges to water treatment processes and environmental remediation. The use of coagulation-flocculation represents a facile and efficient way of removing charged particles from water. The formation of stable colloidal flocs is necessary for floc aggregation and, hence, their subsequent removal. Aggregation occurs when these flocs form extended networks through the self-assembly of polyelectrolytes, such as the amine-based polysaccharide (chitosan), which form polymer “bridges” in a floc network. The aim of this overview is to evaluate how the self-assembly process of chitosan and its derivatives is influenced by factors related to the morphology of chitosan (flocculant) and the role of the solution conditions in the flocculation properties of chitosan and its modified forms. Chitosan has been used alone or in conjunction with a salt, such as aluminum sulphate, as an aid for the removal of various waterborne contaminants. Modified chitosan relates to grafted anionic or cationic groups onto the C-6 hydroxyl group or the amine group at C-2 on the glucosamine monomer of chitosan. By varying the parameters, such as molecular weight and the degree of deacetylation of chitosan, pH, reaction and settling time, dosage and temperature, self-assembly can be further investigated. This mini-review places an emphasis on the molecular-level details of the flocculation and the self-assembly processes for the marine-based biopolymer, chitosan.

## 1. Introduction

Studies on the use of polysaccharides and their removal efficacy toward various waterborne contaminants has been reported. This review focuses mainly on the use of chitosan and its derivatives to facilitate the coagulation-flocculation process for the removal of cationic and anionic species from water. 

The use of biopolymers as flocculants has been increasing in popularity due to the demand for substances that are more environmentally benign and lower cost [1]. Chitosan is an amine-based polysaccharide that fits both of these criteria because it is a readily available biopolymer derived via deacetylation of chitin, the second most abundant polysaccharide in nature after cellulose. Chitosan is a linear polysaccharide comprised of monomers of 2-amino-2-deoxy-d-glucose connected by α-1→4-linkages. The synthetic utility of chitosan relates to the amino group (–NH_2_) at the C-2 position, which can be protonated near its pK_a_ (~6.5) [2,3] to yield cationic polyelectrolyte species in acidic media. At sufficiently alkaline pH, the hydroxyl groups can be deprotonated to give negatively-charged species. The amino and the hydroxyl groups (particularly C-6) can be further modified to give ampholytic chitosan species. Synthetic modification of chitosan affords greater versatility as a flocculant by allowing it to bind with cationic and anionic species leading to removal from solution.

The removal of charged contaminants can be achieved by a wide variety of methods, but one of the simplest and most versatile approaches involves coagulation-flocculation. Coagulation occurs by firstly reducing the charge repulsion of the contaminant species by neutralizing the charges, allowing the particles to associate; resulting in microfloc formation. Thereafter, chitosan may act as a bridging species to nucleate microfloc networks to form macroflocs, which subsequently aggregate and settle out [4,5].

A greater understanding of the mechanistic action of coagulation-flocculation leads to an enhancement of factors that influence the process, such as minimizing sludge formation. The three-dimensional structure of chitosan is key to elucidating the mechanistic action. The structure and morphology of chitosan is dependent on several factors, such as the available functional groups, the degree of deacetylation, molecular weight and the ionization state of the functional groups (pH effects). By examining how a change in these factors affects the efficacy of contaminant removal, it can be inferred how such factors affect the coagulation-flocculation process. A higher level of removal indicates that destabilization of the system was achieved by the reduction of charge repulsion; whereas, a lower level of removal indicates greater charge repulsion and colloidal stabilization.

This review aims to compare and contrast the effects of modified or unmodified chitosan either as a singular coagulant or as a coagulant aid in mixed systems for the removal of charged species from aqueous environments. We also examine various wastewater systems where chitosan has been used as a flocculant and the operating parameters of the coagulation-flocculation process. Overall, the various mechanisms involved relate to the role of self-assembly in the coagulation-flocculation process of chitosan and its modified forms. More importantly, this mini-review provides an overview of the structure-function relationship of chitosan relevant to coagulation-flocculation processes by relating the physicochemical properties of chitosan and the variable removal of contaminants from wastewater systems. We anticipate this overview will lead to further research and development of such coagulation-flocculation processes whilst furthering the long-term goal of developing systems with greater efficiency, lower cost, and sustainability.

## 2. Use of Chitosan in Coagulation-Flocculation

### 2.1. Chitosan as a Coagulant

**Figure 1:**
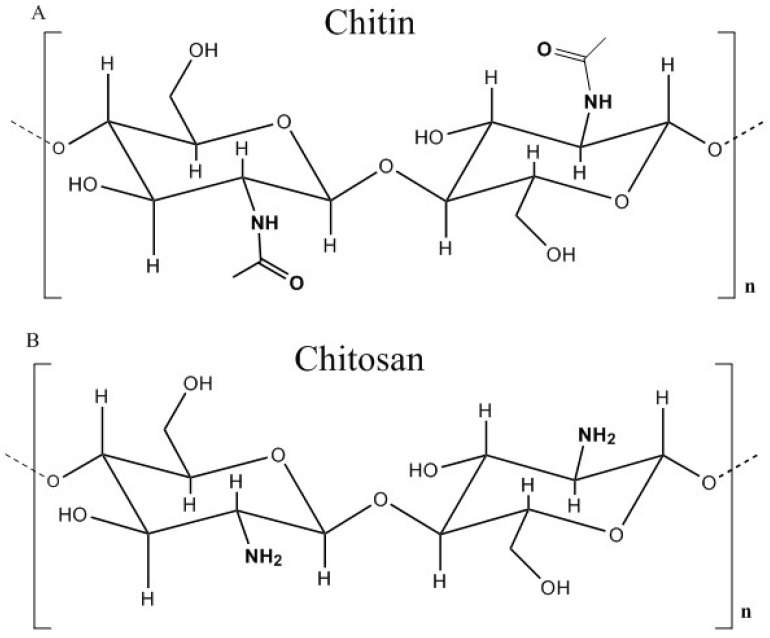
Structure of (**A**) chitin and (**B**) chitosan in its fully deacetylated form, where n represents the degree of polymerization. Redrawn from [2] Hejazi, R.; Amiji, M. Chitosan-based gastrointestinal delivery systems. *J. Control. Release* **2003**.

**Figure 2:**
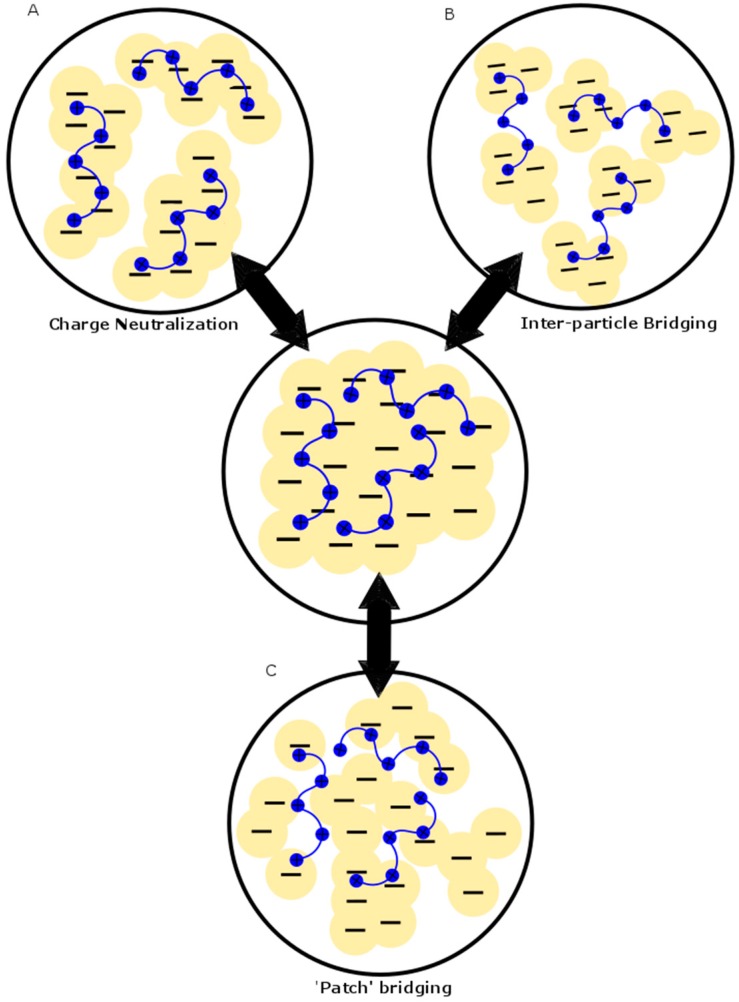
Diagram showing the different mechanisms involved in coagulation-flocculation: (**A**) inter-particle bridging; (**B**) charge neutralization; and (**C**) “patch” adsorption. Redrawn with permission from [4] Li, J.; et al. Optimizing coagulation and flocculation process for kaolinite suspension with chitosan. *Colloids Surf. A Physicochem. Eng. Asp.* **2013**.

Chitosan (see Figure 1) has been used as a single-component coagulant in various systems, such as clay suspensions [4,5,6,7,8,9,10,11,12,13], turbid waters [14,15,16] and various industrial effluents [17,18,19,20]. The key role of chitosan as a coagulant was reported to occur by a charge neutralization process (see Figure 2). Other reports indicate that inter-particle bridging occurs in conjunction with charge neutralization. A “patch” mechanism was suggested due to the fact that the positive charges on chitosan are densely concentrated compared to the negative charges in water where neutralization occurs in smaller regions in a step-wise fashion, thus leading to patches of charge neutralization [4]. The latter occurs by lowering the repulsive interactions between similarly-charged particles, which leads to the compression of the electric double layer. This effect leads to the destabilization of the dispersed system [17]. Since chitosan is a linear polysaccharide that possesses positive ionic charge in acidic media, it also works to bridge flocs together by electrostatic binding of several flocs with the opposite charge along different loci of the biopolymer chain. Evidence for charge neutralization was inferred from studies on chitosan dosage effects, where the removal of turbidity [16,17,21], lignocellulosic materials [20], total organic carbon (TOC) [19,22] and color [23,24,25] increased with increasing dosage up to a point followed by a decrease due to the re-stabilization of the dispersed system. The latter is due to an increase in the positive charge to a point beyond the neutralization of the negative charge sites in the treated water, resulting in charge inversion [19].

Overall, the removal of contaminants from water by chitosan was reported to be of high efficiency at optimal dosage conditions. For the removal of anionic dyes at the stated pH conditions, the efficiency was observed to be above 99% for Acid Blue 92 [25] and ~60%–80% color removal for Reactive Yellow 15 at an optimal dosage of 100 mg/L chitosan. TOC removal for the same dye showed an increase in removal efficiency (%) with an increase in chitosan dosage (74% with 150 mg/L chitosan) [23]. Industrial effluents, such as tortilla [6], pulp and winery wastewater [19], generally contain mixtures of contaminants including organic matter, suspended solids and colloidal materials. Thus, the efficiency of utilizing chitosan in the coagulation-flocculation process was tested on several of these classes of contaminants. Turbidity removal efficiency (TRE) for tortilla waste was about 80% (47 mg chitosan per gram waste solid). Winery waste turbidity removal was 94% at a 400-mg/L chitosan dosage, and TSS removal was 81% at the same dosage. In the pre-hydrolysis liquor (PHL) of pulp production [20], many organic components can be found, such as monomer and oligomer sugars, furfural, acetic acid and lignins. The investigation of the removal of these compounds showed that ~4%–25% of the sugars were removed with less than 2.5 mg chitosan per gram PHL waste; furfural removal was between 50% and 55% with 0.5 mg/g chitosan to PHL; acetic acid removal was 10%–14% at 0.5–1.0 mg/g chitosan to PHL dosage, while lignin removal was 30%–40% at 1.5–3.0 mg/g chitosan to PHL. The removal of turbidity from sea water [17] was found to be quite high at lower doses of chitosan (6–60 mg/L) within 95%–98% for the range. The authors noted that greater turbidity removal occurred in saline water (sea water) versus distilled water. Studies on the removal of kaolinite [4] and bentonite [5] model systems with chitosan in both tap and distilled water investigated the effect of ionic strength on coagulation. Overall, the chitosan resulted in a lower turbidity below 10 NTU (nephelometric turbidity units), where it was concluded that ionic strength (high in tap water) played an important role in turbidity removal. It should be noted that factors such as the level of deacetylation (DA) and the molecular weight of chitosan affect the type of removal mechanism, as discussed in the following sections.

### 2.2. Chitosan as a Coagulant Aid

Many studies have been conducted with chitosan in the presence of other species, where it acts as a coagulant aid. In many cases, chitosan is used along with traditional coagulants to reduce the amount of coagulant required. The mechanism of action is similar when used singularly. However, much of the charge neutralization occurs on negative sites with metal cations and protonated sites of chitosan, depending on the pH conditions. Chitosan acts more as a bridge to bind the preformed microflocs into macroflocs that eventually settle out of the system. The use of chitosan as a coagulant aid leads to less sludge formation, lower cost and lower residual metal toxicity due to the lower usage of metal salts [1]. Since alum is already used for water treatment industrially, the addition of chitosan as an auxiliary coagulant is a simple process that allows for a reduction in the amount of alum required. For the removal of turbidity from sea water [17], the combined use of alum and chitosan proved highly effective at reducing turbidity from 10,000 NTU down to 10 NTU with a dosage of 5 mg/L chitosan and 13.5 mg/L alum. The use of alum or chitosan alone led to a removal efficacy of 40–50 NTU, revealing the unique combination of this system. The binary chitosan/alum system was used to reduce the turbidity of Keddara’s Dam water [16], where a 97% reduction had the added benefit of forming denser flocs at a dosage of 0.2 mg/L chitosan and 40 mg/L alum. Iranian surface water [26] showed a 98% turbidity reduction using 5.0 mg/L alum and 0.5 mg/L chitosan. These results show that the alum-chitosan combination works well for turbidity removal. This study also looked at the removal of hardness from water (using calcium carbonate) and *Escherichia coli* removal, where the maximum hardness removal was 84.3% in low turbid water (10–20 NTU) with an initial hardness at 204 mg/L CaCO_3_. *E. coli* removal was greater, with a 99.99% reduction. This was attributed to the anti-bacterial effect of chitosan related to its binding affinity to the microbe surface which blocks normal cell function. Not only was the alum-chitosan system used for coagulation-flocculation, but it was also combined with ultrafiltration (UF) [15], where an improved efficiency (85.8%–99.4%) was noted at a pressure of 2 bars. Thus, the combined use of UF with coagulation-flocculation illustrates its modularity and further expands the scope of water treatment applications. Similar trends were observed for the case of ferric salts (for example Fe(III) chloride), which were used along with chitosan to remove arsenate and arsenite species from water [27]. Both proved very effective in water treatment, where 0.5 mg/L chitosan to Fe(III) chloride aided the removal of arsenate (100%) and arsenite (80%).

Chitosan has also been paired with clay particulates, such as kaolin [18], due to the role of clays as good adsorbents. Chitosan works well to settle colloids, such as those formed by kaolinite suspensions, while the kaolin adsorbs ions, such as phosphate. This affords the removal of ionic species that might not form flocs with chitosan individually. By adsorbing onto the clay, the system undergoes subsequent settling with chitosan. It should also be noted that many wastewater systems contain colloids, which are attributed to the presence of silt and clay particles, making further addition unnecessary or minimal. Leiviskä et al. [18] showed an improvement of the coagulation-flocculation of lipophilic extractives in debarking wastewater with the use of kaolin and chitosan. The removal efficacy was most evident for the removal of resin acids. The use of such a binary system showed a 100% removal (60 mg/L chitosan with 2 g/L fine kaolin) of hydrophobic particles, and for lipophilic extractives, 91% were removed using 30 mg/L chitosan with coarse kaolin.

In previous reports, chitosan was used to remove organic and inorganic species. For example, chitosan was used to remove cells and toxins of *Gymnodinium catenatum* [28], a type of algae that affects cultivated fish. A binary system containing chitosan with calcium oxide (CaO) or calcium hydroxide (Ca(OH)_2_) was used due to the formation of less bulky flocs, especially in the case of CaO. The formation of bulky flocs was reported to hamper the separation of supernatant and floc. The binary system (0.3 g/L CaO and 0.075 g/L chitosan) proved to be ineffective in removing extracellular toxins in the supernatant and flocs; whereas, removal of *G. catenatum* cells occurred at higher cell concentrations.

### 2.3. Modified Chitosan as a Coagulant

Chitosan is a positively-charged polysaccharide in acidic media due to the protonation of the amino groups. However, the pH of water and wastewater systems may be neutral or alkaline, where chitosan has limited use due to its poor solubility above its pK_a_. For this reason, studies have investigated the modification of chitosan with anionic functional groups in place of some of the hydroxyl groups to allow for greater dissolution over a wider pH range.

Rojas-Reyna et al. [9] compared modified chitosan grafted with glycidyl-trimethyl ammonium chloride (GTAC) at the site of the amino groups. The GTAC-modified chitosan (GTAC-Ch) was compared with commercial polyelectrolytes for the removal of Blauton clay, which contains a high content of fine particles and is a mixture of kaolinite, illite, montmorillonite and quartz. CH_2_ was positively charged over a wide pH range (pH 3–10), where it was found that GTAC-Ch performed similar to commercial products. Turbidity reduction of the system decreased from 3500 NTU down to 0 NTU over a wider range of polymer per substrate (mg/g).

Wang et al. [11] investigated the use of poly(2-methacryloyloxyethyl) trimethyl ammonium chloride-grafted chitosan (chitosan-*g*-PDMC) on the treatment of pulp mill wastewater. Chitosan-*g*-PDMC removed 99.4% turbidity, 81.3% lignin and 90.7% chemical oxygen demand (COD), which surpassed the results obtained with a commercial flocculant. Both the chitosan-*g*-PDMC and the commercial flocculant were used with aluminum chloride as the coagulant.

Rios-Donato et al. [10] made a simple modification to chitosan by replacing the hydroxyl groups with sulphate to form chitosan sulphate (ChS). It was used to flocculate kaolinite, bentonite and alumina. Although ChS was found to have an insolubility window between pH 5.0 and 8.0, the effectiveness of the system was tied to the pH conditions. The particles removed include kaolinite (~30 NTU), which was optimal at pH 4.5–5.5, bentonite (<10 NTU) at 4.5–7.0 and alumina (<5 NTU) at 6.0–9.0. The structure of the particles influence their removal over the variable pH range since there was minimal electrostatic repulsion between ChS and the particles. The particle removal occurred by the charge neutralization mechanism (see Figure 2) and by precipitation of ChS.

Yang et al. synthesized carboxymethyl chitosan-graft-polyacrylamide (CMC-*g*-PAM) with variable grafting (1:1, 1:3 and 1:5 ratio of chitosan to PAM) and studied its utility in treating kaolin suspensions [29], along with anionic and cationic dyes in a follow-up study [24]. Yang et al. also studied 3-chloro-2-hydroxypropyl trimethyl ammonium chloride-modified carboxymethyl chitosan (CMC-CTA) with varying degrees of CTA substitution with kaolin suspensions [14]. The modified chitosan flocculant had an increased solubility and residual turbidity (%) of less than 5% when tested at pH 4, 7 and 11. The highest dosage of the flocculant required was 16 mg/L at pH 11 for the removal of kaolinite. Dye removal using an anionic dye (methyl orange (MO)) and a cationic dye (basic bright yellow 7GL (7GL)) was studied [24]. The highest level of color removal for MO was found to be 92.9% for CMC-*g*-PAM11 (1:1 ratio of chitosan to PAM) and 95.0% for 7GL, along with CMC-*g*-PAM11. As for the CMC-CTA system, enhanced solubility with salt resistance was shown by its favourable flocculation performance. At the optimal dosage of the flocculant (0.08–0.10 mg/L), the level of removal was high as evidenced by its large transmittance. An investigation of the effect of variable CTA revealed that CMC with greater substitution resulted in greater solubility. Furthermore, high CTA content resulted in improved flocculation performance.

Chitosan was used with montmorillonite to form nanocomposites (CTS/NMMT) [30] for the removal of biological cells of *Microcystis aeruginosa* (MA). In that study, the Box–Behnken response surface model was used to study various factors, such as the weight ratio of NMMT to CTS and agitation time affected the removal efficiency of MA. The highest removal was found to be 94.9% using the nano-composite.

In summary, chitosan proves to be a versatile bioflocculant singularly and in tandem with a coagulant aid with variable flocculation ability, according to the conditions. The “patch” mechanism describes the mode of action of chitosan (see Figure 2) because of the polymeric nature of chitosan with its array of charged functional groups. The presence of charge sites related to ionization of chitosan affords bridging of microflocs and formation of macroflocs.

## 3. Application of Chitosan in Coagulation-Flocculation for Water Treatment: An Overview

### 3.1. Clay Particle Removal

Many studies have used clay particles, especially kaolin, as a model system for the study of coagulation-flocculation efficiency with various coagulants and coagulant aids [4,5,7,8,9,10,11,12,14,26,29,31]. This stems from the fact that clays form relatively stable suspensions, and their surface structures are relatively well defined. It should be mentioned that factors such as the irregular shape and thickness of particles, samples without a narrow size distribution and charges (on particle edges) that vary with pH limit clay systems from behaving ideally [32]. Other clay particles studied include illite and bentonite [5,10], a type of clay consisting primarily of montmorillonite (smectite clay). Blauton clay is a mixture of kaolinite, montmorillonite, illite and quartz have been investigated for such coagulation-flocculation processes [9].

The behavior of clay particle systems for coagulation-flocculation depend on the crystal structure and surface charge properties of the clay. Clay particles consist of varying platelets of Al(III) or Si(IV), coordinated with oxygen atoms and hydroxyl ions. Depending on the nature of the particle and the presence of impurities, other ionic species may be present, such as Fe(III), Mg(II) or Fe(II), and counterions, such as Na^+^, Ca^2+^ or K^+^. The surface and layered structure of clays are generally fixed in composition and do not vary significantly. However, the edges, which lie perpendicular to the faces, may vary especially with the pH environment of the dispersion. Adsorption or dissociation of hydrogen ions on oxides results in surface charge formation. In acidic media, there are excess amounts of hydronium ions that impart an increased positive charge. As the pH rises, the ionizable hydrogen ions dissociate, first from silanol (Si–OH), followed by aluminol (Al–OH) groups at higher pH, where the edge surfaces become more negatively charged [33].

Montmorillonite is a 2:1 clay mineral that falls under the larger category type of smectite, where the unit cell formula [Al_2_(OH)_2_(Si_2_O_5_)_2_] consist of sheets of Al(III) coordinated to oxygen atoms and hydroxyl ions in an octahedral structure surrounded by Si(IV) coordinated with oxygen in a tetrahedral structure. Ion exchange may occur with the Al(III) being replaced by Mg(II) or Fe(II), yielding a clay with a slightly negative charge. Counter-ions, such as Na^+^ and Ca^2+^, serve to balance this charge [33,34]. Illite is also a 2:1 clay mineral that falls under the larger type of mica. The structural arrangement is similar to that of montmorillonite; however, it differs by the types and amounts of unique minerals found within the alternating (Si,Al)_2_O_5_ sheets [34]. Kaolinite clay is a 1:1 clay mineral with the general formula Al_2_(OH)_4_Si_2_O_5_. It consists of alternating sheets of Si(IV) coordinated with oxygen atoms in a tetrahedral structure and Al(III) coordinated with oxygen atoms and hydroxyl ions in an octahedral structure. The uncharged layers are stabilized by van der Waals and hydrogen bonding interactions. Thus, the surface-accessible sites and exposed edges are available for potential reactions [4,34].

Many coagulation-flocculation studies that use kaolin as a model system are reported with chitosan as a single component coagulant aid, in its native or chemically-modified forms. In most cases, the chitosan was effective at removing kaolin. Kinetic studies show fast settling of kaolin suspensions within ten minutes of settling. The effect may relate to the formation of dense macroflocs with the chitosan system through the bridging mechanism (see Figure 2). Studies on the zeta potential (ξ) of kaolin have shown the system to be negatively charged over a wide pH range (2–10) [4,10]. This allows kaolin to be electrostatically attracted to the sites of chitosan with positive ξ-values at pH values below the pK_a_ of chitosan. At higher pH values, re-stabilization of the dispersion is possible because chitosan and kaolin are both negatively charged, leading to an increased electrostatic repulsion. Modified chitosan, such as chitosan-*g*-PDMC, may possess positively-charged groups at alkaline pH due to the presence of grafted quaternary ammonium functional groups. The presence of such quaternary positive groups overcomes the negative charge repulsion that otherwise occurs between kaolin and unmodified chitosan [11]. Blauton clay with 60% kaolinite is very similar to kaolin, which is mainly comprised of kaolinite, and reveals similar coagulation-flocculation behavior [9].

Bentonite, mainly comprised of montmorillonite, was also studied, where its zeta potential (ξ) showed a consistent and slightly negative charge across the pH range observed (pH 2–8), much less negative than kaolinite. The negative value of ξ affects the coagulation-flocculation properties, especially with respect to the variable pH in this study [5]. There was particle removal with chitosan sulphate occurring over a pH range of 4.5–7.0, a wider range compared to kaolinite (4.5–5.0). With the reduced negative charge on bentonite as the pH becomes more alkaline and as chitosan becomes less protonated, there will be lesser repulsion than for kaolinite, which affords greater coagulation-flocculation to occur [10]. The foregoing illustrates that variable clay structure affects the efficiency of the coagulation-flocculation process along with the solution chemistry of the system.

### 3.2. Removal of Turbidity from Sea and Surface Water

Chitosan has been used extensively for the removal of turbidity, especially in surface and sea water. The biodegradable nature of chitosan, reduced sludge output, and its relatively low material cost makes this biopolymer an ideal choice for water treatment. Chitosan has been studied as a coagulant aid because it is easy to add to an already existing treatment regime that involves traditional coagulants, such as aluminum sulphate (alum). Alum usage has fallen out of favor due to its relatively large dosage levels, which produce large sludge volumes, and the causal link of aluminum to health issues such as Alzheimer’s disease [27].

The removal of turbidity from Keddara’s dam water showed that the use of chitosan is a facile way to avoid the disadvantages mentioned above. The turbidity of the water was determined to be low at 7.81 NTU, and the pH was slightly basic at 8.1; and the chitosan-alum system displayed a 97% removal. It was noted that the sulphate species display an effect on the sorption properties of chitosan. Support provided by Van Duin and Herman’s hypothesis indicate that chitosan forms large aggregates in the presence of sulphate, which are easily precipitated, and may relate to a higher molecular weight in the presence of sulphate [16].

Saline water obtained from the Red Sea from the beach at Yanbu Industrial City, Saudi Arabia, was studied using chitosan singularly and in mixtures containing alum or iron(III) sulphate. The system was sand-filtered after coagulation-flocculation. Turbidity for the sample was also low at 1.7–2.3 NTU, but it was adjusted to 50 NTU to mimic the turbidity of rain and surface runoff when the pH was 8.15. The results showed that smaller doses of chitosan (6–60 mg/L) worked better than the higher dosages (180–360 mg/L). The dose profile is consistent with a charge neutralization mechanism, where chitosan starts to re-stabilize the turbid system after neutralizing the ionic species. Turbidity removal reached 98% for chitosan only; whereas, the efficiency for chitosan as a coagulant aid ranged from 75%–95%, where a higher level relates to the presence of alum. From the information provided on the chemical and physical properties of the water samples, the water contained many ionic species and an electrical conductivity of 50 mS/cm^3^. An increased ionic strength resulted in greater coagulation-flocculation efficiency that was related to an open conformation of chitosan through self-assembly. This is consistent with greater numbers of contact points between the particles, which enhance coagulation-flocculation, along with the compression of the electrical double layer through charge neutralization (see Figure 2). After filtration, the turbidity was 0.47 NTU with chitosan alone and 0.43 NTU with chitosan and iron(III) sulphate. Thus, better results were obtained with chitosan alone (without metal salt) due to its ability to undergo particle bridging. However, the effect may relate to an excess positive charge due to Fe^3+^ and chitosan in sea water, causing an increase in turbidity that would not occur when using chitosan alone [17].

Chitosan in conjunction with alum was again used to remove turbidity from surface water, which came from Maringá, Brazil. It was processed further using ultrafiltration (UF). The pH of this water was 8.17 and contained ionic species, where the turbidity was the highest at 240 NTU. The use of the two processes together at pressures of 1 and 2 bars was very efficient at a 99.9% turbidity reduction. Coagulation-flocculation treatment prior to filtration was intended to reduce the problems related to natural organic matter (NOM), such as membrane fouling, flux reduction and inferior effluent quality. The results showed that the chitosan-alum system reduced harmful pathogens, such as *E. coli*, by almost 100%. Unfortunately, there was greater membrane fouling at 2 bars that was offset by the higher permeate flux. This facile two-step process was shown to yield drinking water of high quality in a reliable fashion [15].

### 3.3. Anionic and Cationic Dye Removal

The removal of dyes by coagulation-flocculation was investigated using cationic and anionic dyes. Szygula et al. looked at the removal of an anionic dye (Acid Blue 92; AB92) using chitosan alone [25]. Akdemir also looked at the removal of an anionic dye (Reactive Yellow 15; RY15) using chitosan alone [23], while Yang et al. studied the removal of both an anionic (Methyl Orange; MO) and a cationic dye (Basic Bright Yellow 7GL; 7GL) [24]. AB92 was removed very efficiently (~99%) by chitosan. The optimum molar ratio between dye and amine groups of chitosan was determined because it was inferred that the removal mechanism was due to charge neutralization and inter-particle bridging (see Figure 2) between the cationic amine groups of chitosan and the three anionic sulfonic groups of the dye. The calculated stoichiometry was 3:1 for the chitosan: dye system. The effect was consistent with the chitosan chain bridging the three sulfonic groups of the dye via electrostatic attraction, where the stoichiometry was determined from the mole ratio of dye to the amine groups [25].

The removal of RY15 (chitosan dosage 100 mg/L) showed an optimal removal at 80%, which is slightly less than AB92 (chitosan dosage 90 mg/L) [23]. Both dyes possess sulfonic groups, but AB92 has three, with bulkier phenyl rings attached, and RY15 has two with a more linear shape. MO was removed at an optimum dosage of around 90% by chitosan alone (180 mg/L dosage), and it has one sulfonic group and a linear shape. RY15 and MO possess a similar molecular structure, where the extra sulfonic group of the dye allowed for a lower dosage of chitosan. The structure of AB92 has a bulkier phenyl group with a disc-like shape along with the presence of more negatively-charged groups. AB92 had the highest removal with the lowest dosage among the three dyes. It is important to note that the pH at which the coagulation-flocculation takes place is of key importance since the dyes have differing pK_a_ values and a pH-dependent charge. Chitosan is positively charged when the pH lies below its pK_a_ (5.5–6.5) value, along with the degree of deacetylation.

The cationic dye, 7GL, was removed using the chitosan-*g*-PAM and also efficiently removed by chitosan-*g*-PAM11. The latter is among the most porous of the grafted polymers, while 95% removal was observed using a 160-mg/L polymer dose. This flocculation occurred at alkaline pH conditions ranging from 9–13, with an optimum value at pH 11. This pH condition would restrict the tertiary amine groups on the dye and amine groups on the polymer from being positively charged, while allowing the carboxyl groups to be negatively charged [24].

### 3.4. Industrial Wastewater Treatment

Industrial wastewater often contains many different species depending on the industry. The pulp [20], winery, olive mill [19] and tortilla industry [6] effluents were examined to determine the effectiveness of chitosan as a coagulant or as an aid in complex wastewater systems. Lipophilic extractives in effluent from a Finnish pulp mill included fatty acids, sterols, steryl esters, triglycerides and resin acids (pimaric, sandaracopimaric, isopimaric, palustric, levopimaric, dehydroabietic, abietic and neoabietic acids) [18]. The effluent of another pulp mill, located in Eastern Canada, was studied for the removal efficacy of chitosan toward monomer and oligomer sugars, furfural, acetic acid and lignins [20]. Both olive oil mill and winery effluents were tested for turbidity removal, total suspended solids (TSS) and chemical oxygen demand (COD) [19]. Finally, tortilla industry waste or nejayote was subjected to flocculation with chitosan to reduce turbidity [6].

The lipophilic extractives mentioned above were reduced using chitosan with either fine or coarse kaolin. Overall, it was found that the addition of kaolin to chitosan allowed for an effective removal (87%–91%) of lipophilic extractives. In particular, resin acid removal was enhanced, while fatty acids were not removed efficiently among the various extractives mentioned above. The enhanced removal was attributed to the adsorption onto kaolin particles. The pH after flocculation was in the range of 4.2–5.0, with most functional groups (mainly carboxylate groups) protonated. This condition resulted in less electrostatic repulsion, since chitosan is protonated, allowing bridging of the kaolin flocs with the adsorbed extractives [18].

The removal of waste products from the Canadian pulp mill involved chitosan of either high or low molecular weight (HMW or LMW), where these biopolymers showed variable removal efficiency. Thus, the molecular weight dependence of chitosan exists for the coagulation-flocculation process, where the molecular weight had a smaller impact on the level of the removal (%) relative to the dosage of chitosan, especially for HMW chitosan. The removal of oligomer sugars proved to be greater than the removal of monomer sugars. The removal of oligomer sugars was attributed to entanglement with chitosan, as compared to the monomer sugar species. Removal of oligomer sugars requires partial entanglement for effective removal. Furfural had a maximum removal of 55% that was ascribed to hydrogen bonding with chitosan, which also has a fairly low initial concentration in the PHL. Acetic acid had a much lower removal at 13%. Hydrogen bonding of chitosan contributes to the formation of complexes in the PHL system, according to the removal of acetic acid. Acetic acid had a greater initial concentration relative to furfural, while lignin removal was 40% with the use of chitosan [20]. Although lignin does not have a well-defined molecular structure, the presence of abundant hydroxyl and carboxyl groups result in ionization, especially at higher pH values. The removal of lignins with cationic chitosan displayed favourable binding, as described above. Since LMW chitosan had slightly higher removal, it is likely that the relative size of the lignin species was related to chitosan. For the HMW chitosan, the removal of lignin was slightly less and may be due to the longer chains and bridging with lignin particles. Perhaps entanglement of the long chains caused a slight decrease in the removal efficiency.

Both olive mill and winery effluents were analyzed for turbidity, TSS and COD removal. The winery effluents had lower initial loading of turbidity, TSS and COD. The use of chitosan caused a slightly higher turbidity removal for olive mill waste; whereas, winery waste showed greater removal (%) for TSS and COD. Unfortunately, both effluents have variable components based on many factors and were not fully analyzed to determine the composition. The organic matter content relates to the presence of phenols in both effluents, where the olive mill wastewater had higher levels of phenols. The pH was 4.4 for olive mill wastewater and pH 6.8 for winery effluent [19].

The removal of turbidity from *nejayote* was found to be highly efficient at 80%. The *nejayote* system bears similarity to olive mill and winery effluents, containing many organic compounds and represents a very complex system. The turbidity removal with chitosan shows that overall, the waste was negatively charged, as evidenced by ξ measurements. The positively-charged chitosan biopolymer enables flocculation by charge neutralization and inter-particle bridging (see Figure 2). Many organic compounds, such as oligomer phenols found in the effluents mentioned above, possess negatively-charged groups due to the presence of carboxylate anions, revealing the effective removal by chitosan [6]. Industrial effluents tend to contain colloidal species due to their complex nature where chitosan was shown to be effective at forming flocs with such dispersed particles. Studies have shown that clay particles relate to the source of the colloidal particles in the effluent and provide more efficient settling of flocs [18]. Thus, chitosan is very useful for a wide range of industrial wastewater treatment, as will be shown by other studies reported herein.

### 3.5. Organic Cells and Biomaterial Substance Removal

The use of chitosan to remove organic species was examined by looking at the removal of algal cells of *G. catenatum*, and the saxitoxins released were also monitored to determine the removal efficacy by flocculation with CaO-chitosan from the supernatant and flocs [28]. *M. aeruginosa* and the microcystins (MC) released from these cells were removed using chitosan-montmorillonite nano-composites [30]. The results revealed that chitosan enabled removal of the cell components, but not as efficiently as single-component systems, such as CaO or Ca(OH)_2_. Thus, the presence of the divalent Ca^2+^ ions enabled floc formation and settling through charge neutralization, similar to positively-charged chitosan and aluminum sulphate, with comparable efficiency to chitosan. This indicates that the *G. catenatum* cells are negatively charged, and the saxitoxins released from this algal bloom underwent flocculation by the binary system. The CaO-chitosan system was not suitable for the removal of the toxins from the supernatant and flocs. This occurred because the saxitoxin structure contains multiple –NH groups and other positively-charged groups that were repelled by the positively-charged binary system [28].

In a study of *M. aeruginosa* and its microcystin (MC) toxins, the removal of the cells was very effective, at 94.9%. The cells possess a negative charge which allowed for the charge neutralization mechanism to occur with the chitosan portion of the composite, where netting and bridging were also suggested, as evidenced by the re-shaped cell flocs. Montmorillonite usually has a negative surface charge due to the many hydroxyl groups on the clay surface. However, it is not likely that this effect would be responsible for the removal of the cells, while it does aid in the fast settling of the flocs. The MC removal was studied by monitoring both intracellular and extracellular microcystin-leucine-arginine (MC-LR). It was found that the CTS-NMMT first formed a protective layer around the cells. This layer was damaged and caused the cells to rupture and release intracellular MC-LR over time. When the extracellular MC-LR concentration was measured, it showed a slight increase that seemed to be caused by leakage of the intracellular MC-LR; however, the MC-LR was adsorbed by the composite. It was reported that chitosan undergoes degradation, which led to cell lysis and the release of the intracellular MC-LR. Since the composite was posited to be responsible for the removal of the MC-LR, especially the montmorillonite fraction, the abundance of –NH groups on the MC-LR species may be protonated, which accounts for the negatively-charged montmorillonite neutralization and settling process [29].

### 3.6. Arsenic Removal

**Figure 3:**
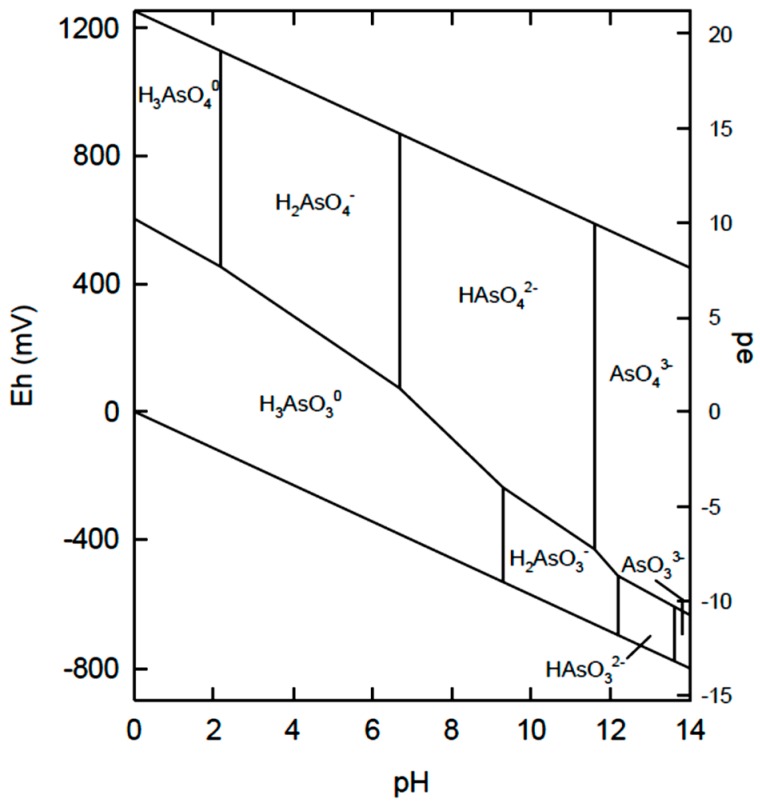
E_h_–pH diagram of arsenic [34] Smedley, P.L.; Kinniburgh, D.G. Sources and behaviour of arsenic in natural water. In *United Nations Synthesis Report on Arsenic in Drinking Water*, **2005**; www.who.int/water_sanitation_health/dwq/arsenicun1.pdf (Date accessed 1 July 2016).

The literature on arsenic removal by coagulation-flocculation with chitosan is sparsely reported [27,33,34,35,36,37,38,39,40]. In general, typical examples of coagulation-flocculation studies have focused on inorganic arsenic species, either arsenate (As(V); HAsO_4_^2−^) and/or arsenite (As(III); H_3_AsO_3_) species [27]. The more oxidized As(V) species (arsenate) tends to be found in surface waters. Arsenite is oxidized to arsenate aerobically, so As(III) will be prevalent in anoxic areas, such as in groundwater environments. In terms of toxicity, the arsenite species are more toxic and mobile over arsenate, where either inorganic form are more toxic than the organic forms of arsenic [36]. Other arsenic species are known based on E_h_-pH diagrams (see Figure 3), where E_h_ is the voltage potential and pe is defined according to electron activity (a_e_), where pe = −log_10_ (a_e_).

Studies on the removal of As(III) are less common than those of As(V), mainly due to the fact that arsenite is readily oxidized to As(V) by aeration. The leading cause of arsenic poisoning in humans is from contaminated drinking water, which tends to contain more arsenate relative to arsenite, due to aeration of surface waters [27]. From the E_h_–pH diagram, it is noted that arsenite (H_3_AsO_3_) generally occurs as the nonionized form over a broad pH range. Thus, H_3_AsO_3_ is more difficult to remove since electrostatic attraction is a key step for coagulant-based removal.

Coagulant aids have been studied in conjunction with Fe(III) salts for the removal of As(III) and As(V) species. Hesami et al. [27] investigated the removal of As(III) and As(V) with Fe(III) and chitosan as the coagulant and aid, respectively. Optimal removal of As(III) (80%–100%) was achieved at pH 7 using Fe(III) at a dosage of 60 mg/L, instead of chitosan as a bioflocculant. However, the addition of chitosan resulted in a smaller dosage of Fe(III). Studies on As(V) showed the same trend, but to a lesser degree, where a 5%–10% drop was observed upon chitosan addition.

**Figure 4:**
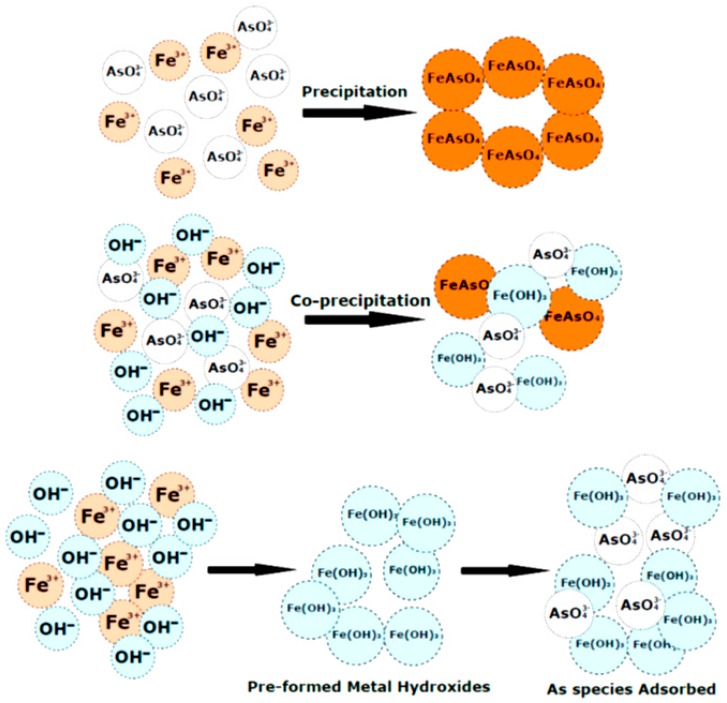
Mechanisms of arsenic removal using metal ions.

The reduction in Fe(III) usage caused a corresponding decrease in residual Fe(III) after coagulation. This is advantageous since increased levels of Fe(III) in water are less aesthetically pleasing in taste and smell [39]. Baskan et al. [35] used the Box–Behnken design to study optimal parameters for the coagulation of arsenate. Above pH 6, it was determined that arsenate removal was about 100% at Fe(III) levels above 30 mg/L. It was suggested that soluble arsenic species are converted to insoluble reaction products through three major steps: (1) precipitation of iron arsenate solid (FeAsO_4_); (2) co-precipitation of arsenic species along with solid metal hydroxides via inclusion, occlusion or adsorption; and (3) adsorption of soluble arsenic species onto pre-formed solid hydroxide precipitates (see Figure 4) [33,34,35]. The primary contribution of chitosan related to the use of lower levels of Fe(III) due to the bridging of flocs between iron and arsenic. Since the pH lies above the pK_a_ of chitosan, precipitation would occur with the arsenic species and Fe(III) hydroxides from solution, where binding occurs through dispersion forces rather than electrostatic attractions. Arsenite species had a noticeable drop in removal due to competition between arsenite and chitosan for Fe(III). Arsenite and chitosan are nonionized at this condition, where favourable association occurs via dispersion forces.

Fewer reports using coagulation-flocculation processes for the removal of organic arsenic (organoarsenicals) are available. Organoarsenicals often contaminate aquatic environments through their use as pesticides and herbicides. Organoarsenicals also form via methylation of inorganic arsenic through bacterial and fungal action. Biomethylation of arsenate may yield monomethyl arsenate (MMA) or dimethyl arsenate (DMA), where such species have been reported in a California lake and a river system in Spain [36]. Hu et al. studied the removal of these two organic species and compared it to arsenate removal using common coagulants: Fe(III) chloride, aluminum chloride and polyaluminum chloride. As with other arsenate studies [35,40], iron(III) performed better than aluminum, where the removal efficiency (%) varied as follows: As(V) > MMA > DMA. This trend led to the conclusion that an increase in methylation hindered the removal of As species, and may relate to variable water solubility and charge effects described above. The ionic binding of As species onto the surface of the Fe/Al hydroxide illustrate the key mechanism of As removal of As in Figure 4 [36].

**Figure 5:**
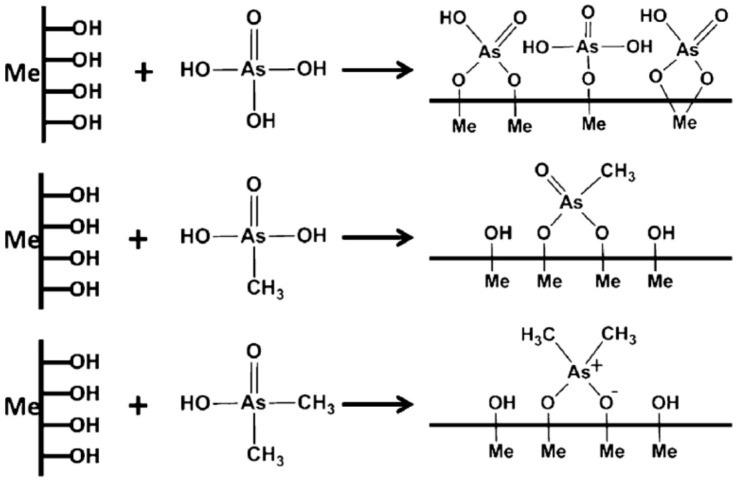
Interaction mechanism between methylated arsenate (As) and Fe/Al (Me) hydroxide during coagulation. Reproduced with permission from [36] Hu, C.; et al. Coagulation of methylated arsenic from drinking water: Influence of methyl substitution. *J. Hazard. Mater.* **2015**.

Figure 5 illustrates that more inhibited binding occurs between the As species and the hydroxide functional groups when more methyl groups are present. Arsenate was postulated to bind as monodentate, bidentate binuclear, or bidentate mononuclear complexes. The presence of methyl groups instead of –OH on arsenate contributes fewer available binding sites. Thus, binding affinity with arsenate exceeds MMA and MMA over DMA. Furthermore, the addition of methyl groups led to increased steric hindrance, which further inhibits binding. Chitosan was evaluated for the removal of DMA to evaluate its uptake. Use of Fe(III) alone results in a 32% removal of arsenate; whereas, the presence of 0.5 mg/L chitosan resulted in an increased removal to 38%. Further addition of chitosan caused a greater removal below 20%. Chitosan did not enhance DMA removal significantly, which may have been partly due to the pH (7.80) conditions of the system. Accordingly, DMA is negatively charged, while chitosan remains uncharged since the pH exceeds the pK_a_ (chitosan), with minor Coulombic attraction between the two species. However, the solubility of the chitosan decreases as the pH increases, where the precipitation of chitosan leads to the adsorption of DMA bound with Fe(III) hydroxides [33,35,40]. Above 0.5 mg/L chitosan, there is a more pronounced drop in DMA removal (%), which may relate to competition between chitosan and Fe(III). Chitosan is a relatively large biopolymer that presents an increased amount of adsorption sites to DMA, which may bind more with Fe(III) and reduce the uptake of DMA.

The foregoing results highlight the versatility of the coagulation-flocculation method using chitosan. For the majority of these systems, colloidal particles were used in their pristine form or integrated into the systems. The mode of action of chitosan was similar to a polyelectrolyte for the destabilization of the colloid by compressing the electric double layer. In the case of systems without colloidal particles, such as As(III), coagulation was aided by the formation of colloidal or solid particulates. A decrease in the solubility of chitosan due to pH would likely result in the removal of contaminant species by precipitation. In all cases, the bridging effect of chitosan plays a role by enhancing the removal of species from water due to its large contact surface area.

## 4. Effect of Operating Parameters of the Coagulation-Flocculation Process

### 4.1. pH Effects

pH is a key factor in coagulation-flocculation processes according to the studies presented above. pH affects biopolymer structure and hydration of the coagulant-flocculant system, along with the chemical nature of the species undergoing removal from the aqueous phase. In addition, pH has a significant effect on the dosage of the coagulant-flocculant system due to the structural changes that occur due to ionization effects.

Chitosan is a cationic biopolymer in acidic media due to the presence of abundant amine groups. The typical pK_a_ (chitosan) is ~6.5, depending on its degree of deacetylation. At pH values below the pK_a_, chitosan is positively charged [3] and is not water soluble above its pK_a_. Thus, pH influences the charge state of chitosan and its overall solubility, as well as its efficacy in flocculation processes. For example, kaolinite clay particles tend to be negatively charged over a wide pH range [4,10], while chitosan is protonated and serves as a suitable flocculant due to the potential of favourable electrostatic interactions with kaolinite particles in acidic media for the removal of turbidity. A rise in pH above the pK_a_ of chitosan results in a reduction of ionization and water solubility. In turn, this alters the flocculation efficiency, either by lowering the removal efficacy or the requirement of a higher dosage of chitosan to work effectively. pH is a key parameter that affords variable chitosan water solubility and flocculation efficiency at different solution conditions.

### 4.2. Dosage Effects

The use of chitosan in place of other traditional coagulants, such as aluminum sulphate (alum) and ferric chloride is advantageous, partly due to its biodegradability and non-toxic nature. However, another advantage related to the use of chitosan concerns its lower dosage requirements when compared against traditional metal salt coagulants [7,17]. An effective dosage of chitosan relates to many factors, such as temperature, pH, the identity of the coagulant-flocculant and the nature of the waterborne contaminant.

Many studies [18,19] have highlighted the fact that above a certain dosage of chitosan, re-stabilization of colloidal dispersions and reduced removal of the contaminant species may occur. The foregoing relates to an excess of positive charge due to excess levels of chitosan [7,17,19]. This indicates that coagulation occurs by the charge neutralization and patch mechanism (see Figure 2). In some cases, higher dosages are needed for optimal flocculation to occur. For instance, the higher initial turbidity of the olive mill waste required a higher dosage of chitosan for optimum flocculation [19]. The effect may relate to the biopolymer chain length, where a more coiled configuration exists and relates to restricted accessibility of the protonated amine groups to enable effective charge neutralization or bridging [4,6,14]. Dosage requirements vary when coagulants, such as alum, are used with the chitosan, especially for modified chitosan. For example, chitosan-*g*-PAM11 showed a lower dosage when compared to unmodified chitosan to achieve similar removal efficiency [24]. The effect is due to the role of grafting which affords a higher MW for chitosan-*g*-PAM11 along with an increased solubility profile in water via the addition of polyacrylamide.

### 4.3. Mechanical Effects

The mechanical aspects of coagulation-flocculation are important, especially for determining the optimal removal efficiency. The mechanism of coagulation-flocculation can be described by two stages: (1) agitation (mixing speed and time) and (2) settling [29,41,42]. The first stage allows for rapid mixing of the coagulant with the dispersion and formation of the flocs, and the second stage allows floc formation with suitable settling of solids from the system. The shortest time that these mixing events can occur is considered optimal since the process demands cost effectiveness. Rapid mixing speeds and short times for settling often yield poor results as indicated by a low removal (%) of contaminants. In some cases, agitation is separated into two stages: fast and slow mixing. The fast stage allows rapid dispersion of coagulant particles and the second stage allows for contact between the particles to form and to maintain floc formation. In some cases, flocs break apart where smaller particles re-disperse at high mixing speeds [43].

Zhang et al. studied the flocculation of kaolinite using chitosan-*g*-PAM at mixing speeds ranging from 100–250 rpm with an agitation time of 10 min. The results showed that 150 rpm was optimal in summer and 100 rpm was optimal in winter. Agitation times were shown to be at the lower end of the mixing range, suggesting that floc breakage may occur at higher speeds or the particles do not have sufficient contact time to form larger flocs before the motion of the liquid pulls them apart. They studied the settling/sedimentation time, and optimum sedimentation was observed at 20 min [44]. Chitosan-CTA-*g*-PAM was used to flocculate kaolin, where the sedimentation time was shown to reach a steady state for all flocculant dosages, except at the lowest and two higher dosages by 20 min. These conditions may result in slower settling due to insufficient flocculant dosage for adequate flocculation (lowest) and a re-stabilization of the dispersion with excess flocculant (highest) [30]. Reed pulp suspensions were flocculated with a chitosan-nanoparticle SiO_2_ system, where the contact and agitation time was shown to be less than 6 min. The authors suggested that flocculation had reached a pseudo-equilibrium state by 1 min and that maximum flocculation may be independent of contact time [44]. CTS-NMMT complexes were used to flocculate *Microcystis aeruginosa* cells, where the agitation time was studied. At an optimal dosage, the agitation time falls between 16 and 50 min [29]. Thus, the contact time for particles and settling time require shorter time intervals when using chitosan as a flocculant. A faster settling time is expected when larger flocs are formed, since they would have greater mass. Multi-functional polymers with many contact points to bridge the flocs would likely result in a shorter contact time due to the high surface area, especially compared to non-polymeric materials.

### 4.4. Temperature Effects

As with any chemical processes, temperature is a key factor that influences the process. Most coagulation-flocculation processes take place at ambient temperature because they occur on a large industrial scale. In temperate countries where the change in season alters the ambient temperature of the treated water, variable temperature studies enable the determination of the optimal conditions such as dosage, according to the temperature effects. Variable temperature studies of model systems are required in order to achieve industrial scale processing, since thermodynamic and kinetic factors which govern such processes can be estimated. This information enables new coagulants, flocculants and contaminants (simple and complex) to be evaluated for efficiency with minimal bench work; thereby saving time and reducing costs.

Variable temperature studies show that temperature affects the dosage level of chitosan required for efficient coagulation-flocculation. The trend shows that as the temperature increased, a lower dosage of chitosan was required [14,41,44], as shown by the greater transparency of the supernatant with increased temperature. Temperature also affects the system in two ways. Firstly, elevated temperature causes the viscosity of the liquid to decrease, which allows contaminant particles to move more freely and faster. This leads to more collisions and an increased chance of particle aggregation. Secondly, the conformation of the polymer chain becomes less coiled and more extended, which allows more accessibility for interactions and bridging with the contaminant particles [14,30].

The factors explored in this section are important to obtaining the maximum removal of contaminant. By optimization, these factors can be manipulated to enhance the coagulation-flocculation process. Factors bearing direct influence on the morphology of chitosan, such as pH, are of particular importance, since the structure and charge distribution of ionic groups influence the polymer structure and morphology.

## 5. Effect of Chitosan Morphology

### 5.1. Molecular Weight

Li et al. [4] studied the effect of the MW of chitosan and its role on the flocculation properties. The MW was reported in units of kilodaltons (kDa). Chitosan (CS) (232 kDa) was used along with DC2 (168 kDa), DC4 (9.5 kDa) and DC6 (1.5 kDa) in this study, where DC refers to the degradation condition used to obtain the chitosans with varying MW. An increase in MW of chitosan corresponds to an increase in chain length, where various chitosan materials were studied in distilled water (DW) and tap water (TW) at an initial dosage of chitosan of 0.10 mg/L and pH = 5.0.

Overall, there was a good removal efficacy of the variable MW values of chitosan in DW. CS had low residual turbidity in TW, while other types of chitosan performed poorly in TW, shown by high residual turbidity. All systems settled rapidly in both DW and TW, where settling was accomplished within 10 min. With the exception of DC2, DC4 and DC6 in TW, the residual turbidity was below 5 NTU. In TW, the residual turbidity decreased as the MW increased.

Clearly, the performance in TW differed from DW in a noticeable way. Coagulation-flocculation reportedly occurred via different mechanisms depending on the MW of the chitosan. High MW chitosan employed the electrostatic patch mechanism, while moderate MW adsorbed by inter-particle bridging and charge neutralization, while low MW chitosan was adsorbed by charge neutralization alone (see Figure 2). Additionally, the low ionic strength of TW (as evidenced by ξ-values [4]) contributed an increase in intermolecular hydrogen-bonding, as compared to DW.

The slight changes in mechanistic action with the MW dependence of chitosan was related to two factors: chain length and the configuration of the biopolymer in water. The increase in the chain length allows for greater availability of positive charges for patches of charge neutralization to occur with the colloidal particles. Shortening of the chain would decrease the amount of bridging facilitated by chitosan. A more extended or open configuration in DW increased the accessibility of the available positive charges.

The chitosan chain conformation was affected by the higher ionic strength of the suspension in TW compared to that of DW and was attributed to electroviscous behaviour due to electrostatic repulsion of the ions on the biopolymer chain. Investigation of the stability of kaolinite in TW showed that the electrical double layer was compressed at increasing ionic strength, resulting in differing aggregation behaviour of kaolinite compared with DW [45] due to a stabilizing effect on the colloid.

### 5.2. Effects of the Degree of Deacetylation

**Figure 6:**
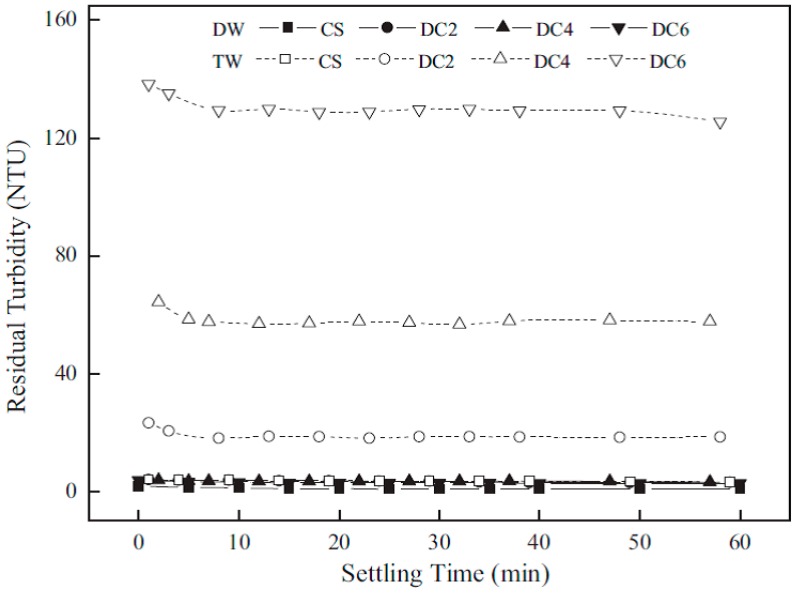
Colloidal stability was assessed through sedimentation tests of kaolinite flocculated with chitosan with different degrees of MW. Chitosan dosage 0.10 mg/L, initial turbidity of kaolinite suspension 200 nephelometric turbidity units (NTU) and pH 5. Reproduced with permission from [4] Li, J.; et al. Optimizing coagulation and flocculation process for kaolinite suspension with chitosan. *Colloids Surf. A Physicochem. Eng. Asp.* **2013**.

**Figure 7:**
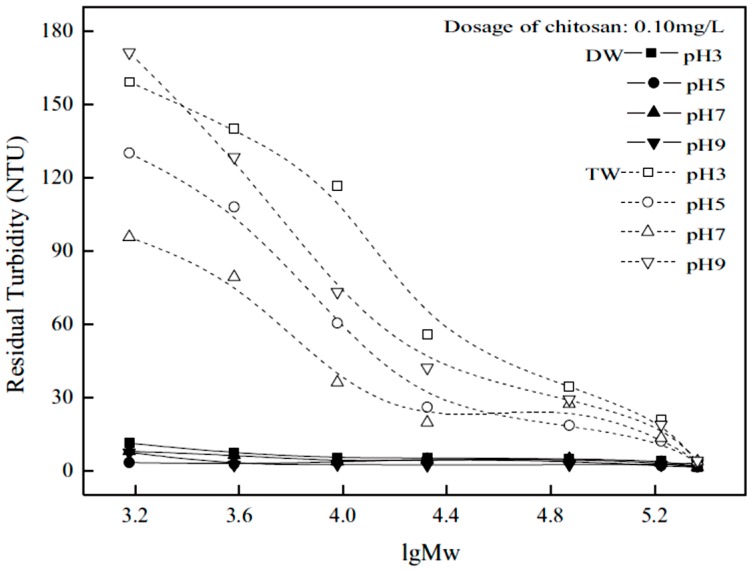
Influence of the logarithmic molecular weight (lgM_W_) and chitosan dosage on the coagulation and flocculation of the kaolinite suspension at different pH in demineralized water (DW) and tap water (TW). Initial turbidity of kaolinite suspension 200 NTU, settling time 5 min. Reproduced with permission from [4] Li, J.; et al. Optimizing coagulation and flocculation process for kaolinite suspension with chitosan. *Colloids Surf. A Physicochem. Eng. Asp.* **2013**.

There are various levels of deacetylation of chitosan for use in the removal of colloids. Li et al. [4] investigated the effect of colloidal stability with various chitosan materials with variable degree of deacetylation (DD): 95.2% (CS), 83.2% (NACs1), 69.7% (NACs2) and 54.6% (NACs3), and its effect on colloidal stability, where NACs refer to N-acetylated chitosan. Figure 6 and Figure 7 show the experimental results of colloidal stability for kaolinite suspensions in TW and DW.

In general, the residual turbidity was less in the DW compared to that in TW, except in the case of NACs3. Chitosan with variable DD performed differently in the two types of water. In DW, the order of performance was CS > NACs1 > NACs2 > NACs3. By contrast, the following trend was observed in TW: NACs3 > CS > NACs2 > NACs1. In all cases for DW, except NACs3, the flocs settled quickly (within 10 min) and remained fairly stable over the time at pH 5 with a chitosan dosage of 0.10 mg/L. NACs3 had the highest residual turbidity in DW (>5 NTU), while the other systems were below 4 NTU.

The short settling times observed indicate that stable flocs of colloidal particles were formed since stable floc networks can aggregate into bigger networks and settle out. The authors suggested that variable colloidal stability related to the use of the different DD, according to the mechanisms shown in Figure 2. A high DD implies a high charge density with different polysaccharide chain conformations. Both effects lead to stabilization of the colloid, but by different mechanisms; namely, charge neutralization and inter-particle bridging.

It was suggested that fast settling implies that both mechanisms occur at the same time. This is plausible since chitosan with the highest DD is the biopolymer that settles rapidly and displays the lowest residual turbidity among samples. A greater amount of positive charge aided neutralization of the negative charges of kaolinite, affording colloidal destabilization through charge neutralization.

A dosage of 0.10 mg/L was used to study colloidal stability where an increase of DD in DW led to a decrease in residual turbidity. Thus, an increase in ionization (from an increase in the number of amino groups) led to greater positive charge on chitosan that aided the neutralization of the negative charges of kaolinite. In TW, the opposite was true, but the effect was less pronounced. This seems to imply that colloid stabilization occurs by increasing the DD in TW. This effect was accounted for by the authors according to an increased negative charge on kaolinite due to ligand exchange from the anions present in the TW. An increase in negative charge may cause a slight stabilization of the colloid due to a similar amount of positive charge on chitosan, resulting in less charge neutralization compared with the results in DW.

Selected results are summarized in Table 1, where the nature of the contaminant varies from dissolved to suspended solids, such as colloidal clays, anionic and cationic dyes, biological cells and several other wastewater systems. This table provides an overview of the versatility of chitosan as a coagulant or coagulant aid.

The pK_a_ of unmodified chitosan is ~6.5. As the pH decreases below pH 6.5, the amino groups become increasing protonated with greater positive charge. Above pH 6.5, the amino groups are deprotonated leads to reduced positive charge as pH increases. pH also affects the surface charge of kaolinite due to ionization of –OH groups found on the edge of the kaolinite surface. In acidic solution, the kaolinite is positively charged due to addition of H^+^ ions. As the pH increases, the protonated sites are ionized, and adopt less positive charge at higher pH. In general, chitosan performed well at pH below 9.0 since chitosan has a slight positive surface charge at pH values near and below its pK_a_. This results in charge neutralization of the kaolinite particle. The lowering of the pH results in less negative surface charge on kaolinite due to protonation of the basic groups.

The morphology of chitosan and other related coagulant-flocculant systems play a key role in such self-assembly removal processes. The contact between the functional parts of the polymer and contaminant is necessary for efficient removal due to the role of complex formation. The structure of contaminant species play a role in maximizing favourable associative interactions. In the case of chitosan, the positive charges and large surface area are crucial to its effectiveness. By increasing the degree of deacetylation and MW, chitosan becomes more positively charged and adopts an extended conformation; both of which lead to enhanced coagulation-flocculation ability, in accordance with the charge and surface area effects (see Figure 2).

## 6. Conclusion

Chitosan as an abundant biopolymer that has been used quite extensively in water treatment, especially as an adsorbent and in coagulation-flocculation processes. It is a versatile material due to its utility as a coagulant aid and a biopolymer flocculant. The availability of variable functional groups can be achieved by grafting and other synthetic modification. The use of chitosan for contaminant removal is extensive, ranging from the removal of clays, industrial waste, TOC, color, anionic and cationic species. The latter is possible through the modification of chitosan with anionic groups. Factors affecting the removal efficacy are dosage, pH, temperature, agitation time and speed, settling time and the MW and DD of chitosan. Among these factors, dosage and pH (external factors) and MW and DD (internal factors) greatly affect the removal efficiency when using chitosan as a flocculant or coagulant aid. Modification of chitosan by varying the MW and/or DD of the biopolymer enables lower dose requirements and a wider solubility window across variable pH conditions.

The use of chitosan or the other polymers that form polyelectrolyte complexes (PECs) will enable further modification of optimum parameters for coagulation-flocculation [46,47,48]. Further probing of the kinetics and thermodynamic parameters is crucial to the understanding of the self-assembly of chitosan into flocs and the further formation of dense flocs. The latter are advantageous because they contain less water and are easier to remove. Dynamic light scattering (DLS) may serve as a useful probe to achieve this goal by providing information on the real-time floc formation and particle size distribution [49,50,51]. The use of a “one-pot” system [52] can also be used to study the kinetics and thermodynamics of a coagulant-flocculant system in situ. The “one-pot” method affords simplification in cases where comparative kinetics can be estimated using a semi-permeable membrane barrier. This method affords variable temperature studies and the determination of the thermodynamic parameters of coagulation-flocculation processes. We anticipate that this review will contribute to the advancement of coagulant-flocculant systems with improved properties for the treatment of wastewater systems.

## Figures and Tables

**Table 1:** Removal efficacy for various coagulant-coagulant aid systems.

Coagulant	Coagulant Aid	Contaminant	Percentage Removal	Chitosan Dosage	Reference
Chitosan	–	Tortilla waste turbidity	80%	47 mg/g Chitosan to solid waste	[6]
Aluminum chloride (1017 mg/L)	Chitosan-*g*-PDMC	Pulp mill waste turbidity	99.4%	17.8 mg/L (pH 7.1)	[11]
Chitosan	–	Sea water turbidity	95%–98%	6–60 mg/L	[17]
Chitosan	Kaolinite (2 g/L)	Lipophilic extractives	91%	60 mg/L	[18]
Chitosan	–	Reactive Yellow 15 dye	60%–80%	100 mg/L	[23]
Chitosan	–	Furfural	50%–55%	0.5 mg/g Chitosan to PHL	[20]
CMC-*g*-PAM11	–	Basic Bright Yellow 7GL dye	95%	160 mg/L (pH 11)	[24]
Alum (5 mg/L)	Chitosan	Iranian surface water turbidity	98%	0.5 mg/L	[26]
Iron(III) chloride (30 mg/L)	Chitosan	Arsenate species	100%	0.5 mg/L (pH 7)	[27]
Chitosan	CaO (0.3 g/L)	Extracellular toxins	Ineffective	0.075 g/L	[28]
CMC-*g*-PAM11	–	Kaolin	95%	16 mg/L (pH 11)	[29]
CTS/NMMT	–	*M. aeruginosa*	94.9%	310 mg/L	[30]

PHL = pre-hydrolysis liquor, CMC = carboxymethyl chitosan, PAM = polyacrylamide, PDMC = poly (2-methacryloyloxyethyl) trimethyl ammonium chloride, CTS = chitosan, NMMT = nano-sized montmorillonite.
